# Long-Term Recidivism of Mentally Disordered Offenders Considered “Dangerous to the Public” in Switzerland

**DOI:** 10.3389/fpsyt.2021.639936

**Published:** 2021-04-06

**Authors:** Daniela Schaffner, Michael Weber, Tanya Kochuparackal, Marc Graf, Henning Hachtel

**Affiliations:** ^1^Department of Forensic Psychiatry, University Psychiatric Clinics, Basel, Switzerland; ^2^Faculty of Medicine, University of Basel, Basel, Switzerland; ^3^Office of Corrections, Canton of Zurich, Zurich, Switzerland; ^4^Department of Psychology, University of Konstanz, Konstanz, Germany

**Keywords:** forensic psychiatry, criminal recidivism, major mental illness, long-term follow up study, violent reoffending risk, high-risk offenders, time at risk

## Abstract

**Objectives:** The commissions for risk assessment of offenders dangerous to the public were established in 1995 in Switzerland. The main goal was to reduce recidivism of offenders released into the community by means of identifying high-risk offenders and recommending measures for offender management. This study investigates long-term recidivism data of this high-risk cohort of offenders.

**Methods:** Baseline data included risk assessment of one of the commissions, the type of index offense, and psychiatric disorders according to ICD-10 for the total cohort of offenders examined by the commissions between 1995 and 2009. Criminal records were drawn in 2019 for all offenders from the Swiss Federal Office of Justice.

**Results:** From a total of 147 offenders 35 recidivated within a median time at risk of 9.1 years (31.8%), of which 10 (9.1%) recommitted a severe offense. Within the treatment status, sentences (imprisonment and preventive detention) were compared to court-ordered measures (in- or outpatient court ordered treatment, civil court mandated treatment, vocational training facility). There were no significant differences comparing treatment status, different diagnostic groups, type of index offense and other risk factors. Except of age at release (or relapse), which predicted recidivism with younger subjects showing higher recidivism rates (*p* = 0.014).

**Conclusion:** Our study showed that over a long-term time at risk this high-risk cohort showed a similar recidivism rate as many other studies with different cohorts. With appropriate management recidivism rates in high-risk offenders can be lowered allowing them being consecutively reintegrated into society. The finding that younger subjects have higher recidivism rate was reproduced in this population.

## Introduction

The prediction of recidivism by mentally disordered offenders still is an issue with inherent methodological limitations and severe legal implications. To decide whether the assumed risk profile of an individual is sufficiently reduced to order a release is a difficult decision (1).

Many studies have shown that reoffending or non-compliance with parole supervision are common among mentally ill offenders (9–78%), but the conviction rate for violent crimes is notably lower (10–34%). In these analyses, the median time at risk (TAR) was 6 months to 9 years. Generally, recidivism rates increased with longer observation periods (2–11).

Most of those studies' follow-up times are relatively short, and longer observation periods are needed to optimize knowledge when performing forensic psychiatric assessments and risk assessments (8, 12). Still there is only little knowledge about recidivism in offenders who are convicted of a severe crime. Long follow-up times that permit a longer TAR are needed, especially in this group of patients, where base rates of reoffending are low.

Many studies have shown that unstructured clinical judgment can poorly predict future offenses (13–16). Aegisdottir and Grove showed in their meta-analyses, that statistical prediction techniques are superior to the clinical approach, especially in predicting violence (17, 18). Nevertheless, there are limits to the use of statistical tools when it comes to assessing the individual risk of recidivism, both for scientific and legal considerations: They rely on a fixed and restricted set of risk factors that have been validated for use in specific samples in specific contexts, thus working particularly “on average” across subjects. Assessment of future risk posed by an individual solely based on such tools is therefore limited in its informative value and ethically questionnable, especially if grounded in mostly static risk factors (19).

Focusing on the particular situation in Switzerland in the 1990s, the common procedure for risk assessments (before subsequent mentioned event) was an unstructured clinical approach with its inherent disadvantages. After a sexual murder by a prisoner on weekend leave in Switzerland in 1993 and the following public outcry, the national management of dangerous offenders had to be completely thought over. Several working groups found the current method to ease offenders lacking, which was mostly based on intuitive opinions of involved therapists (20, 21).

A new procedure was established in 1995 to assure more objective, safe, and accurate handling by installing a peer-reviewed process: The so-called “commissions for risk assessment of offenders dangerous to the public.” The respective boards were manned with five to seven people: A judge as chairman, a representative of the prosecution, officials from penal institutions, and a forensic psychiatrist as well as officials from victim protection agency. The main tasks of these commissions are to determine the dangerousness to the public, the risk for relapse of an offender, and to provide the correctional services recommendations for the management of the offender.

The decisions of the boards are based on a structured professional judgment: the “Catalog of Areas of interest for Risk Assessment in high-risk offenders” (CCRA), which in its newest version is titled “Basel Catalog for Risk Assessment” (BCRA). This checklist of so-called “criminogenic areas of interest” was developed by Prof. V. Dittmann in the wake of his personal experience with predicting reoffense and broad expert knowledge (22–24). The BCRA contains criminological, sociological, and specific factors for personality and mental diseases, each in two dimensions: risk and protective aspects.

A recent analysis of national level data on reoffending in Switzerland reported a decrease in the three-year reconviction rate over the last years, resulting in a rate of 35% of general recidivism for the year 2013. A further decrease of the recidivism rate was predicted (estimation: 28% for the year 2020) (25).

This study is a report of rates of reconvictions at a long-term follow-up (median TAR 9.1 years; Range: 1 day−22.9 years) of the special high-risk cohort of patients considered “dangerous to the public” after a severe index offense in Switzerland. The recidivism in different diagnostic groups, type of index offense, and other risk factors are matter of interest as well.

## Materials and Methods

### Study Design

The study is a retrospective case-note analysis of all the 147 male offenders (the four female offenders had already been excluded) assessed by the Bernese Board within the period from February 1995 (when the board was founded) to November 2008 (when this duty was taken over by over-regional boards). According to Swiss regulations, an offender considered dangerous to the public represents an immediate and serious threat to life or physical condition, as well as to mental health for a non-definable group of people (26).

Baseline data was collected in 2009 in the Bernese office for probation and law enforcement. We examined the first (or in certain cases several) written ratings of the BCRA/CCRA of 147 offenders. Psychiatric disorders according to ICD-10 (27), type of index offense, and the assessment of the commission board were collated for each participant. Whether the psychiatric disorder was offense-related, was examined as well.

The BCRA is a structured professional judgment and main instrument of the peer-reviewed process of the commissions. After a revision in 2019, it features a more precise item description and assessment guideline (comparable to the HCR-20 V3) (24). The catalog now consists of the following 12 offense-related factors: (1) analysis of the index offenses; (2) criminal history until the index offense; (3) personality, psychiatric disorder; (4) insight of the offender into his personality or existing psychiatric disorder; (5) social competence; (6) personality specific conflict behavior; (7) workup of the committed crime; (8) general therapeutical efficacy of treatment options; (9) individual treatment options; (10) willingness for therapy; (11) social conditions in case of release; and (12) follow-up development after index offenses (24). Ratings of the BCRA were made in general by several experts of the commission independently to ensure reliability.

In 2019, the update data was collected in the Bernese office of probation and law enforcement. The end of data collection was on November 1, 2019. All data concerning recidivism, newest judgments, and treatments were recorded. Death records as well as migration were considered after consulting the Swiss Federal Statistic Office.

### Objective

Associations between treatment, diagnostic categories, and type of index offense and recidivism were analyzed. This involved comparing the reconviction rates of different subgroups, considering observation periods of different lengths. An important goal was the comparison with general recidivism rates of offenders.

### Measures

Independent variables were the treatment status, offense-related diagnostic category, and index offense category.

Within the treatment status, sentences (imprisonment and preventive detention) were compared to court-ordered measures (in- or outpatient court-ordered treatment, civil-court-mandated treatment, vocational training facility). Offense-related diagnostic categories were made of the main diagnoses, which had no overlap between categories (ICD-10 F2, F65, and F60). No other diagnostic groups could be made because of overlapping F2, F65, or F60 diagnoses or too few cases.

Regarding index offense categories, sexual offense was compared to any other offense, considering that 97.3% of all index offenses were severe offenses (only *n* = 2 offenders committed other than violent offenses as index offense, which was arson with danger to life and limb).

The dependent variable was recidivism, which was reconviction for any crime. The first new offense during TAR was recorded as recidivism. If there had been severe crimes after the first recidivism, they were recorded separately. Offenses within the detention or prison were not counted as recidivism, except for most severe reoffenses (murder or sexual offenses).

Type of crime can be specified as violated article of the Swiss criminal code. Crimes were entitled as severe after the definition of the United Nations Office on Drugs and Crime's (UNODC) international classification of crime for statistical purposes (ICCS) (28). The first four categories (acts leading to death or intending to cause death, acts leading to harm or intending to cause harm to the person, injurious acts of a sexual nature, acts against property involving violence or threat against a person) were classified as severe crimes, although only severe violence against a person was counted in.

TAR was defined as time between release and (a) death (if not recidivated before death—no single case); (b) end of observation period (Nov 1, 2019); (c) relapse (date of recidivism); OR (d) if reoffended with murder or sexual offense before being released; in this case, TAR was set at day 1. Deportation from the country did not end the TAR period. Outpatient court-ordered treatment was considered as TAR.

### Procedure

To examine the relationship between risk factors and time to failure, time-event analysis (Kaplan-Meier survival estimates) and Cox proportional hazard regression were performed. In a first step, the relationship between recidivism and each of the study variables (conventional sentence and treatment measure, category of sexual and other index offenses, F2 diagnoses, diagnosis of personality disorder, and diagnosis of paraphilia) was examined using log-rank tests. In a second step, a multivariate Cox Proportional Hazard analysis including the variables with *p* < 0.2 in the univariate analyses was performed. Lack of power was a major limitation of this study. Conclusions are therefore limited, and multivariate analyses were conducted for exploratory purposes only.

## Results

### Study Population

The initial sample consisted of 147 male and 4 female offenders, which is the total cohort of the patients considered “dangerous to the public” by the commissions. All female offenders were excluded, due to the small sample size. Subsequently, the study cohort included 147 male offenders, of which 110 offenders had been exposed to a TAR. In the following paragraphs, we will focus on offenders exposed to a TAR (*N* = 110).

A majority (60.9%, *n* = 67) of the study cohort had committed a homicide as index offense, 49.1% bodily harm (*n* = 54), and in 30.0% of all cases, the index offense was a sexual offense (rape, sexual integrity, child molestation) (*n* = 33). About 25.5% had committed offenses of robbery (*n* = 28), and 22.7% had committed other offenses of property (*n* = 25). Multiple answers within the different offense categories were possible.

In 26.4% (*n* = 29) of the cases, the criminal court imposed a prison sentence, and 60.9% of the cohort received a psychiatric treatment during or after sentence (*n* = 67), while in 12.7% of the subjects' preventive detention (*n* = 14) was imposed. In 17.3% (*n* = 19) of the cases, the court imposed an inpatient treatment in a forensic psychiatric clinic, while 42.7% (*n* = 47) received treatment in prison. Two cases were imposed according to juvenile criminal law because of minor age at the time of the index offense and were administered to a vocational training facility. At the time of the first assessment through the commission, all participants were of age.

Psychiatric diagnosis according to ICD-10 were diagnosed in 74.8% (*n* = 80) of the samples, and 40.0% of the subject had at least two psychiatric diagnosis (*n* = 44). In the following paragraph, we focus on the diagnoses considered relevant to the index offense (defined so by the commissions).

A majority of 23.9% (*n* = 26) of the study cohort met the criteria of a disorder of adult personality and behavior (F6), thereof 6.5% (*n* = 7) were diagnosed with paraphilia. About 10.2% (*n* = 11) of the cases suffered from mental and behavioral disorder due to psychoactive substance use (F1) with no other offense-related main diagnosis. Schizophrenia, schizotypal, and delusional disorder (F2) were diagnosed in 5.6% (*n* = 6) of the subjects, and 3.7% suffered from an organic, including symptomatic, mental disorder (F0) (*n* = 4). Co-morbidity within the different psychiatric diagnosis was possible.

Two offenders had died within the follow-up period, and 33 (30.0%) had been deported into their home country immediately upon release from custody. Two offenders have escaped the imprisonment and are still on the run.

### Recidivism

There were totally 35 recidivists (31.8%) of which 10 (9.1%) committed severe reoffenses. For further characterization of the study population see Table 1.

**Table 1:** Characteristics of the study population.

	**Missing** **values**	**Non-recidivists** **(*n* = 75)**	**Recidivists** **(*n* = 35)**
**Therapy status**			
Any measure	*n* = 0	*n* = 46 (61.3%)	*n* = 21 (60.0%)
**Diagnoses**			
Paraphilia (offense-related)	*n* = 2 (1.8%)	*n* = 7 (9.6%)	*n* = 0
F2 diagnosis (offense-related)	*n* = 2 (1.8%)	*n* = 2 (2.7%)	*n* = 4 (11.4%)
Personality disorder (offense-related)	*n* = 1 (0.9%)	*n* = 15 (20.3%)	*n* = 11 (31.4%)
**Index offense**			
Sexual offense	*n* = 0	*n* = 25 (33.3%)	*n* = 8 (22.9%)
Violent offense	*n* = 0	*n* = 48 (64.0%)	*n* = 27 (77.1%)
Other	*n* = 0	*n* = 2 (2.7%)	*n* = 0
**Covariates**			
Age at release	*n* = 0	*M* = 40.7 (SD = 11.2)	*M* = 34.4 (SD = 8.7)
Deportation = yes	*n* = 0	*n* = 26 (34.7%)	*n* = 7 (20.0%)

**Figure 1:**
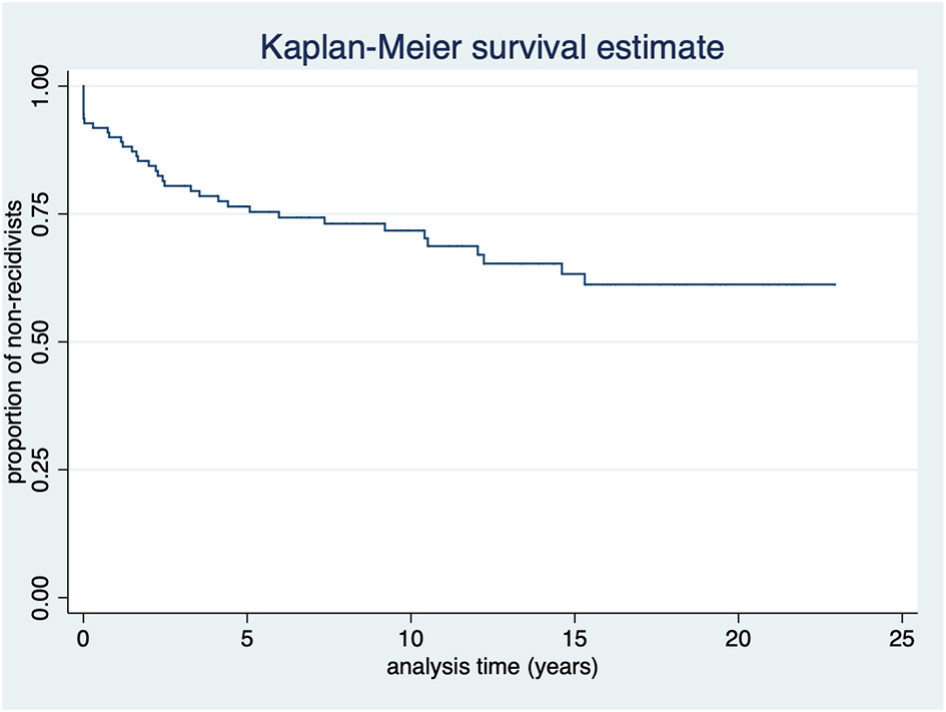
Kaplan-Meier survival estimate of all participants (*N* = 110).

The mean age at first assessment was 34.4 years (SD = 10.9; Range: 19–66 years), the mean age at release or reoffending was 38.7 years (SD = 10.8; Range: 19–69 years), the mean TAR was 9.7 years (SD = 7.2; Range: 1 day−22.9 years; Median: 9.1 years), and the mean time until relapse was 4.0 years (SD = 4.6; Range: 1 day−15.3 years). The Kaplan Meier curve in Figure 1 illustrates the time-recidivism correlation.

**Figure 2:**
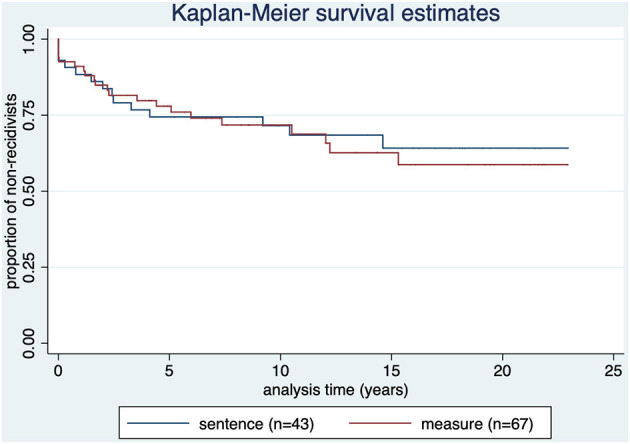
Kaplan-Meier survival estimate by treatment status. *p* = 0.797.

The proportion of non-recidivists over time according to setting (measure *n* = 67, prison *n* = 43) is shown in Figure 2.

**Figure 3:**
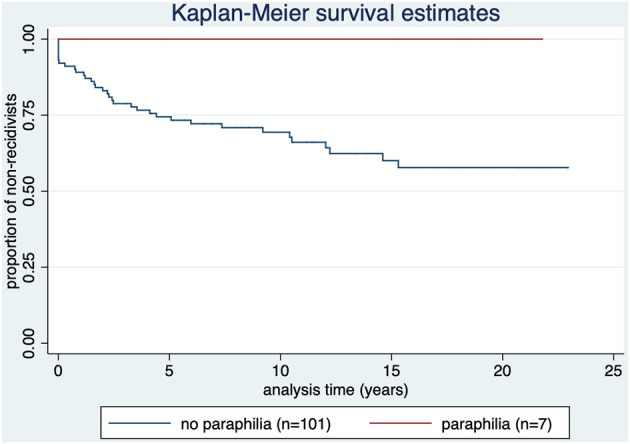
Kaplan-Meier survival estimate by diagnosis of offense-related paraphilia (ICD-10 diagnosis of F65 category). *p* = 0.076.

As shown in Figure 3, none of the subjects diagnosed with offense-related paraphilia (*n* = 7) reoffended.

Four out of six offenders with F2 spectrum diagnosis as offense-related main diagnosis reoffended (66.7%). Three out of the six offenders reoffended before they were released. Time to relapse: *M* = 5.6 (SD = 5.9); median = 5.1; range: 1 day−12 years.

**Figure 4:**
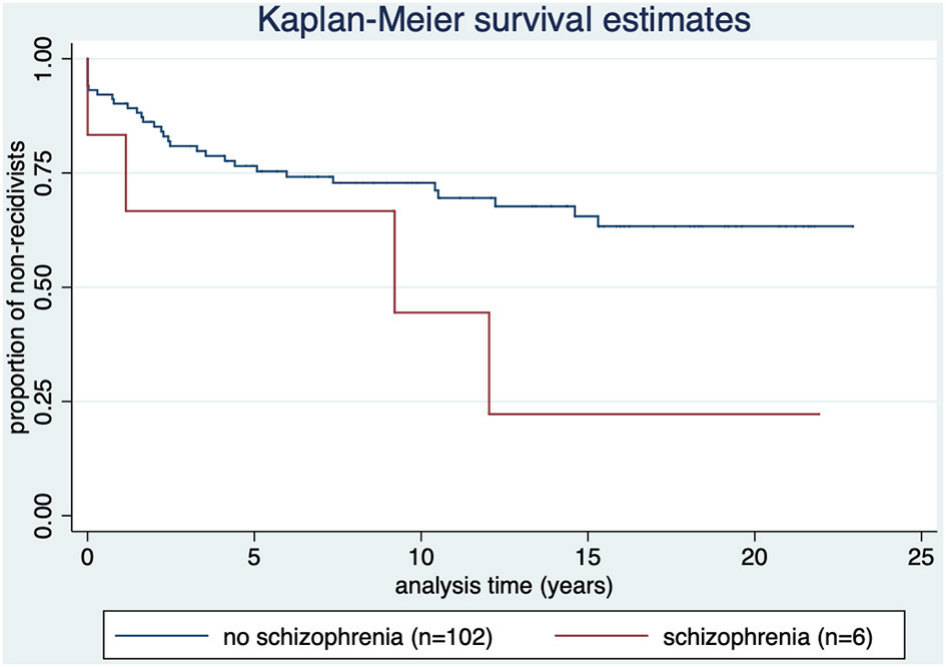
Kaplan-Meier survival estimate by diagnosis of offense-related diagnosis of the F2 spectrum (ICD-10 category F2). *p* = 0.074.

Those offenders reoffending after release (*n* = 3) had a time to relapse of *M* = 7.5 (SD = 5.6); 1.1 year, 9.2 years, and 12.0 years as seen in Figure 4.

**Figure 5:**
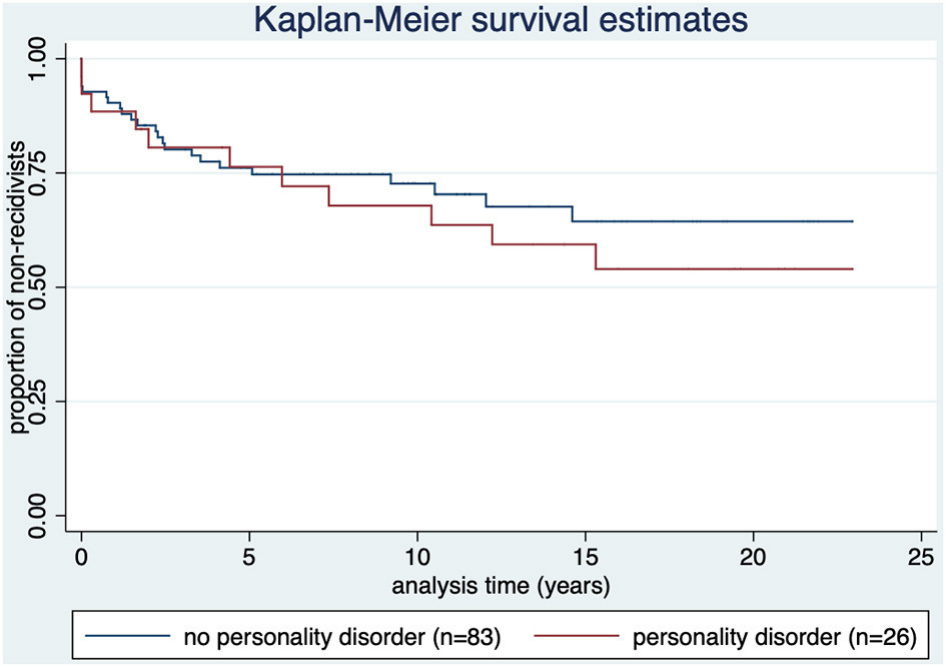
Kaplan-Meier survival estimate by diagnosis of offense-related diagnosis of personality disorder (ICD-10 category F60). *p* = 0.499.

Figure 5 shows that in offenders with personality disorder the recidivism rate was 42.3% (*n* = 11). Time to relapse: *M* = 5.4 (SD = 5.3); median = 4.4; range: 1 day (1 case)−15.3 years.

**Figure 6:**
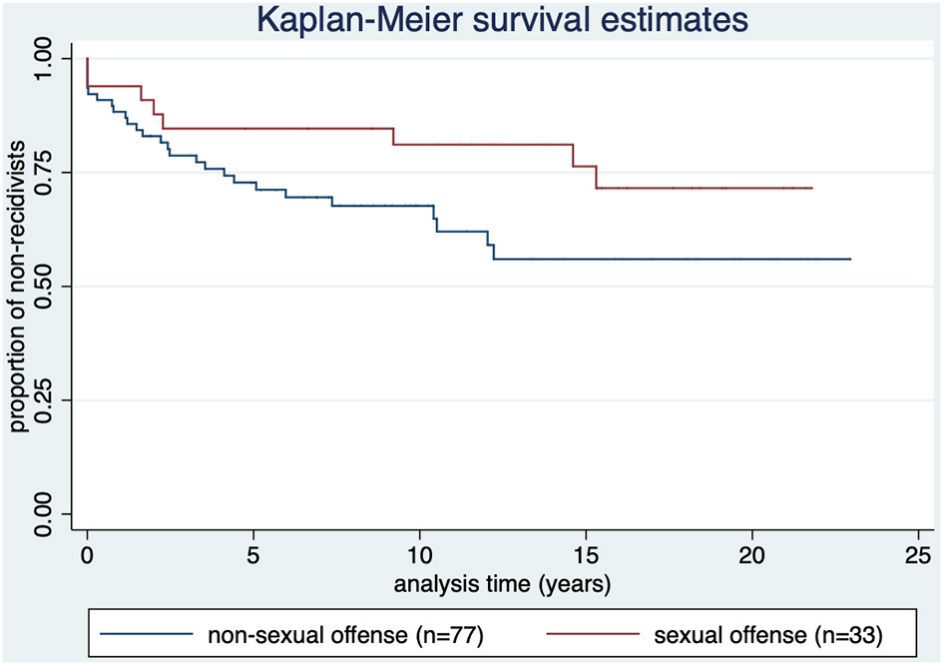
Kaplan-Meier survival estimate by diagnosis type of index offense. *p* = 0.103.

As illustrated in Figure 6, 24.2% of the sex offenders (*n* = 8) reoffended [compared to 35.1% (*n* = 27) of offenders without sexual index offense].

Besides the study variables, age (*p* = 0.014) and deportation status (*p* = 0.084) were examined as additional relevant factors. Age and deportation status as predictors were analyzed as covariates. All predictors that had a *p*-value < 0.2 were included in a Cox regression model. Age at release (or relapse), predicted recidivism with younger subjects showing higher recidivism rates [*B* = −0.05, Wald = 2.45, *p* =0.014, HR = 0.95, 95% CI = (0.91, 0.99)].

Of the 34 deported offenders, 20.4% had a reoffense in Switzerland (*n* = 7). Offenders who had been deported to their home country relapsed less than the other offenders did, although not significantly (*p* = 0.084).

## Discussion

The results on general and specific recidivism as a main treatment outcome in forensic psychiatric treatment are partly inconsistent in the literature and reveal a great heterogeneity of results (29–31). However, mandated treatment was reported as effective on specific recidivism (e.g., sex offender treatment) (32–34). In our study population, therapeutic interventions compared to conventional sentence showed no significant difference (*p* = 0.797) in predicting recidivism. Data of the present sample suggests that offenders with a measure generally had more severe psychiatric diseases, worse risk assessments, and were assumed to pose a higher risk for recidivism. Considering the higher load on risk assessments in the intervention group, the similar recidivism rates of severely disordered offenders to offenders with less severe psychiatric disorder leads to the assumption, that therapy was effective in reducing recidivism rates. Therefore, it is our understanding, that through successful therapeutic interventions, recidivism rates can be reduced (35–37).

This evaluation shows similar reconviction rates as in the general population of offenders in Switzerland and other countries, although recidivism would be assumed to be markedly higher in this high-risk population due to negative selection bias. The surprisingly low level of total recidivism may result from adequate risk management that is appropriate to the respective offender. In particular, an increased proportion of convicted offenders in court-ordered measures seems to be related to a decrease in recidivism (25). In our sample, the proportion of offenders who are sentenced to a measure is higher than in the general criminal population (as there is a preselection of increased risk). But even though it is 60.9%, not all offenders were treated in a measure (some were only detained). This might be an indication that although measures lead to a lower relapse rate on average, the individual assessment of each case is just as important or more important than a “one size fits all” approach, according to which all offenders with a certain basic risk rate are convicted to a measure. Also, we registered fewer or similar severe reoffenses (consisting of severe violent offenses), compared to other studies and meta-analyses (2, 3, 5, 6, 8, 10, 11).

A main target of evaluation and treatment of offenders is harm reduction. In summary, it is our understanding, that through good risk assessment and intervention (psychiatric therapy, measures of law enforcement like social skills training, handling addiction, occupational interventions, and follow-up care), most of the offenders could be rehabilitated, while the minority of ~9% reoffended severely.

Offense-related paraphilia showed less reoffenses than no paraphilia (none at all), although not statistically significant (*p* = 0.076). Sex offenders tend to show lower recidivism rates compared to other violent offenders (albeit not significant) (*p* = 0.103), which is consistent with other studies (38, 39). These results are explainable with the improvement of their risk profile through therapy (six of the seven offenders with paraphilia had court-ordered treatment) and increasing age. Still, the number of unreported reoffenses might be notably higher (40).

According to Hanson, after 10 years of relapse-free rehabilitation, the risk for recidivism of sex offenders matches the risk of the regular population (41). Therefore, after this observation period of median 9.1 years, sex offenders might not be considered as high-risk offenders anymore.

Subjects with an offense-related F2 diagnosis reoffended more often after 5–10 years, although not statistically significant (*p* = 0.074). These reoffenses might have been caused by maladherence due to the diagnosis of schizophrenia, schizotypal, or delusional disorder (F2). Longer recidivism periods would in this case be the result of loosening compliance and aftercare.

There were 12 (11.2%) of cases with F2 diagnosis. In only six of those, the F2 diagnosis was relevant to the index offense and therefore counted into our analysis. The proportion of 11.2% correlate with other analyses (42).

There were relatively few diagnoses of the F1 spectrum as well. In 9 out of 20 cases, there were overlapping with F60 (*n* = 7) or F65 (*n* = 2) diagnoses, which were relevant to the index offense as well. We decided to count those cases to the F60 or F65 group.

Offenders with offense-related personality disorder showed no statistical difference for recidivism compared to subjects with no personality disorder and offenders with paraphilia (p=0.499). It has been shown that an especially antisocial personality pattern is a predictor for violent and also for general recidivism, but not the diagnosis of personality disorder (unspecified). Furthermore, antisocial personality pattern has been shown to be a better predictor in samples not consisting of mentally disordered offenders. Since the underlying data do not allow a differentiation by type of personality disorder, and the sample is largely composed of mentally disordered offenders, this might explain our findings (43).

Offenders, who had been deported to their home country, relapsed less than the other offenders did, although not significantly (*p* = 0.084). Only the relapses in Switzerland are registered, and there is a high probability that reoffenses in other countries are missed. When deported, offenders are generally not administered to imprisonment or therapy.

The effect that younger subjects have higher recidivism rate is well-known (6, 8, 44–46) and was reproduced in our study with high-risk offenders. In line with the mentioned literature, our results suggest age as a major risk factor with high effect size for general criminal recidivism.

### Limitations

A considerable limitation results out of the small study population and substantial lack of statistical power (β- or type-II error), although it is a total cohort assessment. Because of the comparable low base rate of severe crimes, the sample size was limited. This may lead to a biased estimation of base rates; especially the percentages of F2 and F65 diagnoses were low.

Due to the small number of female offenders in the cohort, they were excluded in this study, and we cannot draw any conclusions for female offenders. Further, we did not investigate treatment integrity and did not include the duration of treatment.

Of the 33 offenders who were deported to their home country, only the relapses in Switzerland could be considered. There was no control for execution of the deportation.

TAR may have started earlier when the offenders were still considered to be in in-patient treatment but worked and/or lived in an external facility.

## Conclusion

With appropriate management (evaluation procedure through forensic psychiatric experts and the commissions, measures through law enforcement, court-ordered treatment in a forensic psychiatric clinic, etc.), recidivism rates in high-risk offenders can be lowered, allowing them being consecutively reintegrated into society. Which quantitative influence specific factors have on the outcome was not determinable in this study. This long-term analysis of high-risk offenders shows similar recidivism rates as in a general population of offenders, but with a substantially longer follow-up period. A group of high-risk offenders was identified and administered to therapy, which may have improved their recidivism rates. This study tried to provide base rates for reconviction of paraphilic, offenders with personality disorders, and disorders of the F2 spectrum. Only a younger age was a significant predictor for a higher risk of recidivism.

## Data Availability Statement

The raw data supporting the conclusions of this article will be made available by the authors, without undue reservation.

## Ethics Statement

The studies involving human participants were reviewed and approved by Research Ethics Committee of North-Eastern Switzerland. Written informed consent for participation was not required for this study in accordance with the national legislation and the institutional requirements.

## Author Contributions

DS, HH, MG, and TK designed the study and collected the data. DS took the lead in writing the manuscript, although in close collaboration with and under supervision of HH and MG. MW performed the statistical calculations. All authors provided critical feedback and helped shape design, analyses, interpretation and manuscript.

## Conflict of Interest

The authors declare that the research was conducted in the absence of any commercial or financial relationships that could be construed as a potential conflict of interest.
